# Neuron-inspired CsPbBr_3_/PDMS nanospheres for multi-dimensional sensing and interactive displays

**DOI:** 10.1038/s41377-025-01742-z

**Published:** 2025-01-17

**Authors:** Junhu Cai, Xiang Zhang, Yu Chen, Wenzong Lai, Yun Ye, Sheng Xu, Qun Yan, Tailiang Guo, Jiajun Luo, Enguo Chen

**Affiliations:** 1 National and Local United Engineering Laboratory of Flat Panel Display Technology, College of Physics and Information Engineering, Fuzhou University, 350108, Fuzhou, China; 2 Wuhan National Laboratory for Optoelectronics and School of Optical and Electronic Information, Huazhong University of Science and Technology, 430074, Wuhan, China; 3 Fujian Science & Technology Innovation Laboratory for Optoelectronic Information of China, 350108, Fuzhou, China

**Keywords:** Quantum dots, Imaging and sensing, Displays

## Abstract

Multifunctional materials have attracted tremendous attention in intelligent and interactive devices. However, achieving multi-dimensional sensing capabilities with the same perovskite quantum dot (PQD) material is still in its infancy, with some considering it currently challenging and even unattainable. Drawing inspiration from neurons, a novel multifunctional CsPbBr_3_/PDMS nanosphere is devised to sense humidity, temperature, and pressure simultaneously with unique interactive responses. The carefully engineered polydimethylsiloxane (PDMS) shell enables the reversible activity of the core CsPbBr_3_, serving a dual role similar to dendrites in conveying and evaluating external stimuli with high sensitivity. Molecular dynamics analysis reveals that the PDMS shell with proper pore density enhances the conductivity in water and heat, imparting CsPbBr_3_ with sensitive but reversible properties. By tailoring the crosslinking density of the PDMS shell, nanospheres can surprisingly show customized sensitivity and reversible responses to different level of stimuli, achieving over 95% accuracy in multi-dimensional and wide-range sensing. The regular pressure-sensitive property, discovered for the first time, is attributed to the regular morphology of the nanosphere, the inherent low rigidity of the PDMS shell, and the uniform distribution of the CsPbBr_3_ core material in combination. This study breaks away from conventional design paradigms of perovskite core-shell materials by customizing the cross-linked density of the shell material. The reversible response mechanism of nanospheres with gradient shell density is deeply explored in response to environmental stimuli, which offers fresh insights into multi-dimensional sensing and interactive display applications.

## Abstract (web summary)

Drawing inspiration from neural architectures, the customized cross-linking density in shell materials of perovskite/polymer nanosphere enables advanced multi-dimensional sensing and interaction.

## Graphical abstract

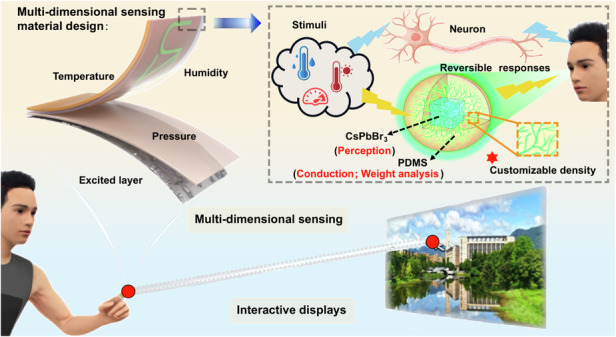

## Introduction

The growing demand for artificial intelligence technologies highlights the necessity for skin-like intelligent sensing devices with enhanced multifunctionality. Transitioning from single-functionality to multi-sensory and multifunction capabilities is crucial for these devices to mimic or exceed human sensory abilities^1^. Enhancing the visualization and interactivity of perceived stimuli will improve the usability of sensing devices, underscoring the significance of developing multi-dimensional sensing materials capable of human communication^2^.

Perovskite quantum dots (PQDs) are versatile materials known for their multiple sensitive and interactive luminescence properties, making them attractive for sensing applications^3–5^. Although PQDs exhibit varying photoelectric properties in response to external environmental stimuli, their strong ionic properties will cause irreversible fluorescence quenching when uncoated^6,7^. Current research has primarily focused on enhancing the stability of PQDs for mainstream applications like displays^8,9^, lighting^10,11^ and solar cells^12,13^, neglecting the need for reversible and multi-sensitive fluorescence responses that are crucial for sensing applications. The challenge lies in designing PQDs that maintain such responses, necessitating material modifications to achieve multi-dimensional sensing capabilities. The single sensing property has been studied, while multi-dimensional sensing characteristics based on a single perovskite material still have a long way to go^14,15^. It emphasizes the importance of designing materials that can adapt and respond reversibly to a variety of stimuli, thereby fulfilling a multi-dimensional sensing role.

Drawing inspiration from neurons, this work designs and demonstrates the perovskite core–shell structured CsPbBr_3_/PDMS nanospheres to address the aforementioned research trends and challenges. Analogous to neurons, the nanosphere consists of a CsPbBr_3_ core and a polydimethylsiloxane (PDMS) shell, with the PDMS shell playing a crucial role similar to neuronal dendrites by conveying and weighting external stimuli to the CsPbBr_3_ core while maintaining the reversible activities for PQDs. The porous PDMS ensures sensitivity and reversibility to environmental stimuli like humidity and temperature, facilitating effective interaction with the external environment while avoiding excessive perturbation to the PQD core structure. By tailoring the crosslinking density of the PDMS shell, the response specificity and selectivity for different stimuli levels can be customized, allowing the nanospheres to perceive precise and wide-range sensing of stimuli. Additionally, the regular pressure-sensitive property is ascribed to the regular morphology of the nanosphere and the intrinsic low rigidity of the PDMS shell, in conjunction with the uniform distribution of the CsPbBr_3_ core material. The innovative design of the multifunctional CsPbBr_3_/PDMS nanosphere holds promise for applications in cutting-edge technology fields such as artificial intelligence and human–computer interaction.

## Results

### Design and preparation of CsPbBr_3_/PDMS nanospheres

**Fig. 1:**
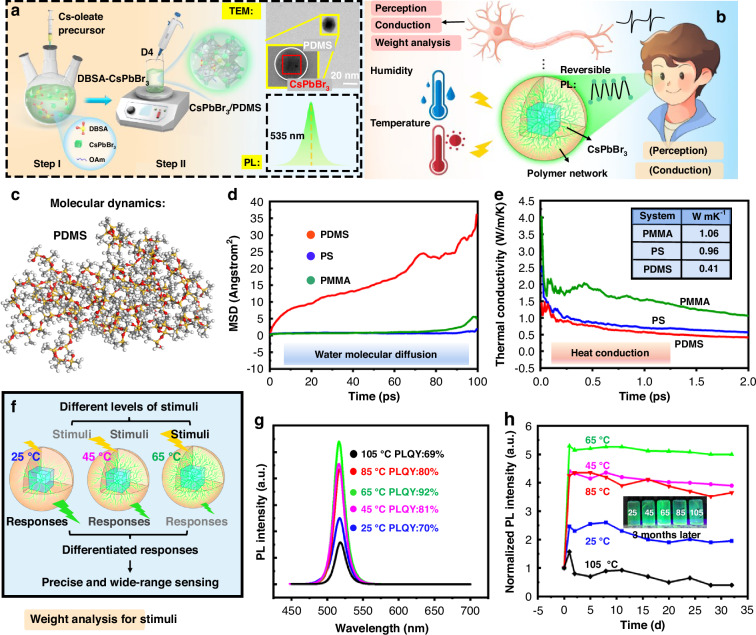
**Design and preparation of CsPbBr**_**3**_**/PDMS nanospheres. a** The preparation flow chart and characterizations of the CsPbBr_3_/PDMS nanospheres. **b** CsPbBr_3_/PDMS design idea drawing. **c** Molecular model of PDMS. Comparison of **d** water molecular and **e** heat conduction behavior of polymers. **f** CsPbBr_3_/PDMS nanospheres with gradient MW. **g** PL spectra and **h** stability of CsPbBr_3_/PDMS nanospheres with gradient MW

The preparation scheme of CsPbBr_3_/PDMS nanospheres is depicted in Fig. 1a. Step I describes the synthesis of dodecyl benzene sulfonic acid (DBSA) ligand-modified CsPbBr_3_ (DBSA-CsPbBr_3_) PQDs via the hot-injection method. Here, the reasons for selecting DBSA as ligands in place of the traditional oleic acid can be mainly attributed to the following two points: Firstly, DBSA furnishes the requisite acidic environment for the ring-opening of D4, serving as a catalyst by ionizing into free sulfonate ions and hydrogen ions within the solution (Fig. S1)^16^. Secondly, DBSA facilitates the robust binding of exposed Pb^2+^ through the formation of stable bonds with SO_3_^−^ groups. Also, it can effectively occupy Br vacancies, thus eliminating the probability of exciton capture^17^. The ligand optimization strategy enhances the lattice stability of the CsPbBr_3_, rendering it less susceptible to irreversible environmental degradation. This enhanced stability is a prerequisite for reversible reactions and a pivotal attribute for perceptual stability in sensing applications. Subsequently, D4 is introduced to the DBSA-CsPbBr_3_, and ring-opening polymerization is conducted at 65 °C to form PDMS (Step II). As depicted in the top right corner of Figs. 1a and S2, the CsPbBr_3_/PDMS nanosphere features a standard mononuclear shell structure. The photoluminescence (PL) spectrum is shown in the lower right corner of Figs. 1a and S3. Given the Gaussian distribution of human visual perception across the green light spectrum, with peak sensitivity at ~535 nm^18^, our research has meticulously engineered CsPbBr_3_/PDMS nanospheres, calibrating their emission wavelength to comply with the laws of visual perception. This targeted design allows for the acquisition of the perceptual results through variations in luminosity or hue, fostering an intuitive and effective visual interaction paradigm with users.

The design of the CsPbBr_3_/PDMS nanosphere as a versatile sensing material draws inspiration from neurons (Fig. 1b). Neurons have the function of perceiving, conducting, and weighting analysis for stimuli, which are essential for information processing. Analogously, a CsPbBr_3_/PDMS nanosphere consists of a CsPbBr_3_ core and a PDMS shell, with external stimuli conveyed to the CsPbBr_3_ core via the polymer crosslinking of PDMS. The PDMS shell, functioning akin to dendrites, is pivotal in maintaining core material’s reversible activity and delivering external stimuli, also with the function of weighting analysis for stimuli (discussed later in Fig. 1f). Similar to the nerve fibers of neurons transmitting electrical information through action potentials^19^, the CsPbBr_3_/PDMS nanospheres visualize changes in light perception, enabling information acquisition through visual stimuli.

Not all perovskite core–shell nanospheres or composites are ideal for multi-dimentional sensing materials. The key is to design core–shell materials with reversible activity, where the protective shell should neither deactivate the perovskite nor render the perovskite material overly stable. Additionally, they should be responsive to diverse stimuli in sensing.

The selection of shell materials is guided by molecular dynamics analysis and experimental observations. We performed comparative analyses of the water and heat conduction behaviors of three common shell coating materials: polymethyl methacrylate (PMMA), polystyrene (PS), and PDMS (Figs. 1c and S4). The simulation results are illustrated in Fig. 1d, e. Regarding water molecules, the water molecular diffusion coefficient of PDMS is significantly higher than that of PS and PMMA, which can be attributed to the dense crosslinking and low porosity of PS and PMMA^20,21^. Consequently, even though PS and PMMA are excellent packaging materials for enhancing the stability of PQDs^22,23^, they may not be optimal for sensing.

Another concern arises regarding PDMS’s higher water molecular conductivity, which could potentially increase contact with water molecules and damage the lattice, causing irreversible quenching. To address this concern, the prepared CsPbBr_3_/PDMS nanospheres were exposed to varying humidity levels between 90% relative humidity (RH) and 40% RH. Stable and reversible fluorescence responses are observed without quenching even under high humidity conditions (Fig. S5a). This suggests that CsPbBr_3_/PDMS nanospheres show promise as materials for humidity sensing.

Additionally, a comparison of thermal conductivity among the polymer materials (Fig. 1e) reveals that the thermal conductivity of PS falls between that of PDMS and PMMA. And the fluorescence reversibility is maintained for perovskite nanospheres coated with PS and PDMS under temperature variations (30 and 150 °C), while the CsPbBr_3_/PMMA composites show limited fluorescence reversibility over time, with fluorescence can not be recovered (Fig. S5b–d). This observation aligns with the simulation results, indicating that higher thermal conductivity in PMMA leads to greater heat accumulation on PQDs. Based on both simulation and experimental results, we tentatively identify CsPbBr_3_/PDMS nanosphere as a promising material for both humidity and temperature sensing.

However, the further design of the nanosphere is essential to accommodate diverse environmental stimuli and effectively respond to a wide range of humidity and temperature levels. By leveraging the adjustable crosslinking density (in analogy with molecular weight (MW)) of the PDMS, we developed a series of CsPbBr_3_/PDMS nanospheres with gradient crosslinking density by varying the polymerization temperatures (denoted as CsPbBr_3_/PDMS@X °C) (Fig. 1f). Details of the nanospheres are provided in Fig. S6. PDMS coating with different crosslinking densities results in varying levels of passivation and protection of CsPbBr_3_. Previous studies have shown that the formation of Pb–O bonds can passivate Br vacancy defects on the CsPbBr_3_ surface, thereby enhancing the optical properties and lattice stability^24^. The nanospheres polymerized at 65 °C, with the highest PDMS crosslinking density, exhibit optimized optical properties and stability (the trend is shown in Fig. 1g, h). Consequently, the CsPbBr_3_/PDMS @65 °C nanospheres are likely to respond exclusively to high humidity and temperature, with low humidity and temperature insufficient to induce fluorescence changes (Fig. S7a, b). The crosslinking density of CsPbBr_3_/PDMS@85 °C and CsPbBr_3_/PDMS@105 °C nanospheres closely resembles that of CsPbBr_3_/PDMS@45 °C and CsPbBr_3_/PDMS@25 °C nanospheres. So, the nanospheres polymerized at 25, 45, and 65 °C are analyzed emphatically due to the impact of polymerization temperature on the CsPbBr_3_. As depicted in Fig. S7c–f, CsPbBr_3_/PDMS@25 °C and CsPbBr_3_/PDMS@45 °C nanospheres exhibit reversible responses at lower humidity and temperature levels. Due to less dense shell crosslinking, they exhibit more pronounced responses to external stimuli, as illustrated in the schematic diagram at the top of Fig. 1f.

In summary, the three types of nanospheres can collectively sense varying levels of humidity and temperature, akin to how dendrites conduct signals based on weight values. Next, the investigations will delve into the reversible mechanism underlying the sensitive and reversible responses of the nanospheres to stimuli.

### Insight into the reversible mechanism of CsPbBr_3_/PDMS nanospheres

#### Reversible response to humidity

**Fig. 2:**
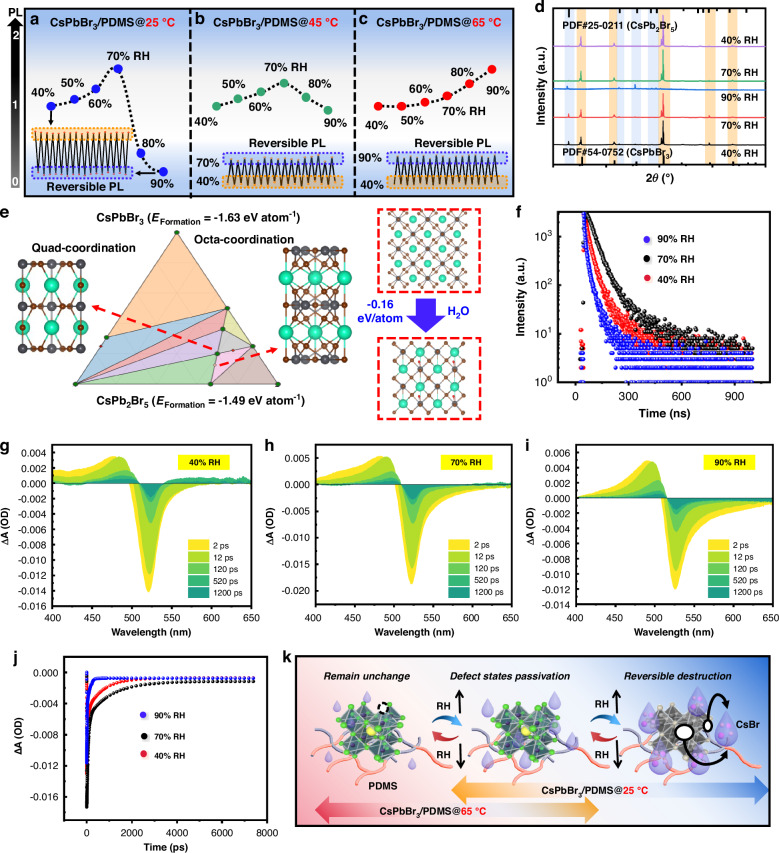
**Reversible response to humidity. a**–**c** Fluorescence changes of nanospheres with humidity. **d** XRD changes of nanospheres with humidity. **e** DFT results of humidity-induced phase transitions of CsPbBr_3_. **f** TRPL changes of nanospheres with humidity. **g**–**i** TA spectra and **j** TA comparisons of nanospheres with humidity. **k** Humidity-sensing mechanism of CsPbBr_3_/PDMS@25 °C nanospheres

The fluorescence changes of the nanospheres with increasing humidity are illustrated in Fig. 2a–c. CsPbBr_3_/PDMS@25 °C and CsPbBr_3_/PDMS@45 °C nanospheres exhibit similar fluorescence patterns, albeit with varying degrees, showing a trend of initially increasing and then decreasing fluorescence with humidity. CsPbBr_3_/PDMS@65 °C nanospheres initially demonstrate fluorescence stability followed by fluorescence enhancement. Importantly, all nanospheres exhibit reversible fluorescence changes in response to humidity variations, with distinctions observed between rising and falling reversibility. Given that the CsPbBr_3_/PDMS@25 °C nanosphere exhibits the most complex fluorescence behaviors and the largest amplitude, it is selected as the focus of this exploration.

The X-ray diffraction (XRD) results of CsPbBr_3_/PDMS@25 °C during humidity changes are depicted in the Fig. 2d. It is observed that at levels of 40% and 70% RH, the nanospheres’ lattice structure remains unchanged, and all the diffraction peaks are well indexed as CsPbBr_3_ (PDF#54-0752). However, at humidity level of 90% RH, the crystalline phase of the CsPbBr_3_ mainly transforms into CsPb_2_Br_5_, with diffraction peaks at 2*θ* = 12.6°, 22.5°, 24.9°, and 29.3°, corresponding to the (002), (210), (202), and (213) planes of CsPb_2_Br_5_ (PDF#25-0211)^6^. This phenomenon suggests that under high humidity conditions, water induces CsBr shedding (CsBr is highly soluble in water), transforming the fluorescent CsPbBr_3_ into non-fluorescent CsPb_2_Br_5_^25,26^. Regarding the observed discrepancies in the diffraction peak intensities during the phase transition, it is likely attributed to subtle changes in atomic positions or bond lengths and angles during the phase transition^27,28^. As an indirect semiconductor, CsPb_2_Br_5_ has a large band gap, which stems from its 2D connectivity character of [PbX_6_]^4−^ octahedrons, with transitions occurring between the X-point of the valence band and the Γ-point of the conduction band. In contrast, CsPbBr_3_ has a 3D structure, in which [PbX_6_]^4−^ octahedrons are connected by sharing the corner, leading to a smaller band gap^5,29^. The primary rationale behind the low luminous efficiency in indirect band gap semiconductors is the diminished possibility of radiative recombination due to their electronic transition mechanism, alongside the potential for concurrent non-radiative recombination processes (Fig. S8).

The water-induced phase transition is corroborated by density functional theory (DFT) simulation results (Fig. 2e). The water molecule adsorption induces the phase transition of CsPbBr_3_, causing Cs to transition from a quad-coordination state in CsPbBr_3_ to an octa-coordination state in CsPb_2_Br_5_ (right part of Fig. 2e). However, this decline in fluorescence is reversible, and when the environment is adjusted to low humidity, the XRD patterns revert to CsPbBr_3_ (Fig. 2d). As humidity decreases, the reduction in water molecules results in the release of CsBr from the water molecules. CsBr, constrained by the PDMS network, then recrystallizes with non-fluorescent CsPb_2_Br_5_ to produce fluorescent CsPbBr_3_, which has been verified in a previous study^29^. In terms of ground state formation energy (left part of Fig. 2e), CsPbBr_3_ represents a relatively stable phase (*E*_Formation_ = −1.63 eV atom^−1^), whereas CsPb_2_Br_5_ is an unstable phase (*E*_Formation_ = −1.49 eV atom^−1^). Consequently, in the presence of Cs and Br ions, the unstable CsPb_2_Br_5_ phase will spontaneously transform into the stable CsPbBr_3_ phase.

The reversible fluorescence decline of nanospheres at high humidity can be attributed to the reversible degradation and recrystallization of the perovskite lattice. While the lattice remains unchanged with low humidity, and the reversible fluorescence increase is likely attributable to dynamic changes in defect states. Our conjecture is verified by time-resolved photoluminescence (TRPL) spectra and ultrafast transient absorption (TA). The PL decay curves (Fig. 2f) are well-fitted using the double exponential equation, and the obtained parameters are summarized in Table S1. Notably, in relation to the long-term radiation recombination process, *τ*_2_ initially increases before decreasing, whereas *τ*_1_ exhibits the opposite trend, indicating the change of non-radiative recombination. The average fluorescence lifetimes are 36.5, 43.7, and 32.3 ns, respectively. More defects will show a smaller fluorescence lifetime^30^. The decrease in defects of CsPbBr_3_/PDMS@25 °C nanospheres under low humidity conditions may be attributed to minimal water contact with the PQDs, which does not alter the lattice structure. Conversely, trace amounts of water (H_3_O^+^ and OH^−^) enhance the crystallinity of CsPbBr_3_, thereby reducing surface defects^31^. However, when the humidity level continues to increase, CsBr shedding induced by water leads to an increase in surface defects, resulting in a shorter fluorescence lifetime. As we have previously analyzed, fluorescence decline at high humidity is associated with lattice degradation.

To further validate changes in defect states and elucidate the exciton trapping process, ultrafast TA tests were conducted (Figs. 2g–j and S9). Bleach recovery at 90% RH is observed to be the fastest, followed by 40% RH, with the slowest recovery observed at 70% RH. Additionally, the bleach recovery dynamics of the samples exhibit double exponential kinetics, with short and long lifetimes. The fitted kinetics for 40% RH show two-time constants of 49.8 ps (*τ*_1_, 7%) and 1670.5 ps (*τ*_2_, 93%), for 70% RH show 49.4 ps (*τ*_1_, 6%) and 2107.2 ps (*τ*_2_, 94%), while for 90% RH show 26.8 ps (*τ*_1_, 12%) and 374.3 ps (*τ*_2_, 88%) with much faster decay kinetic processes. The short lifetime is related to the exciton capture process, while the long lifetime is associated with bounded exciton recombination. A longer exciton lifetime indicates fewer surface trap states in CsPbBr_3_^32,33^. Results reflect that lower humidity levels result in the passivation of water molecules and decreased defects in CsPbBr_3_/PDMS@25 °C nanospheres. Conversely, higher humidity levels induce ion shedding, resulting in increased surface defects^34^.

The effect of humidity on CsPbBr_3_/PDMS@25 °C nanospheres exhibits a biphasic response: initially positive, then negative (Fig. 2k). A small quantity of water molecules passivates the surface defects in PQDs without damaging the lattice structure, leading to fluorescence enhancement. However, as water molecule contact increases, CsBr dissolution occurs, transforming fluorescent CsPbBr_3_ into non-fluorescent CsPb_2_Br_5_, resulting in decreased fluorescence. Crucially, these fluctuations in fluorescence are reversible, primarily due to the bidirectional mechanism underlying the fluorescence changes. When the humidity gradually decreases, the recrystallization phenomenon and defects exposure phenomenon may reappear. Among these, CsPb_2_Br_5_ is a stable, water-resistant, material featuring a two-dimensional Pb–Br framework separated by Cs layers^35^. This unique structure endows the material with enhanced hydrophilic resistance. So the water-resistant CsPb_2_Br_5_ matrix that has been decomposed is difficult for further degradation. CsPb_2_Br_5_ can recrystallize stably with CsBr to form fluorescent CsPbBr_3_, which is pivotal for the stable reversible reactions observed in CsPbBr_3_/PDMS nanospheres.

Inspired by the above, the observed phenomenon of initial fluorescence stability followed by an increase with rising humidity in CsPbBr_3_/PDMS@65 °C nanospheres can be explained: PDMS polymerized at 65 °C provides robust protection for core CsPbBr_3_, preventing direct contact with trace water molecules and resulting in fluorescence stability under low humidity conditions. As humidity gradually rises, trace water molecules come into contact with the PQDs, resulting in surface defect passivation and fluorescence enhancement (Fig. 2k).

#### Reversible response to temperature

**Fig. 3:**
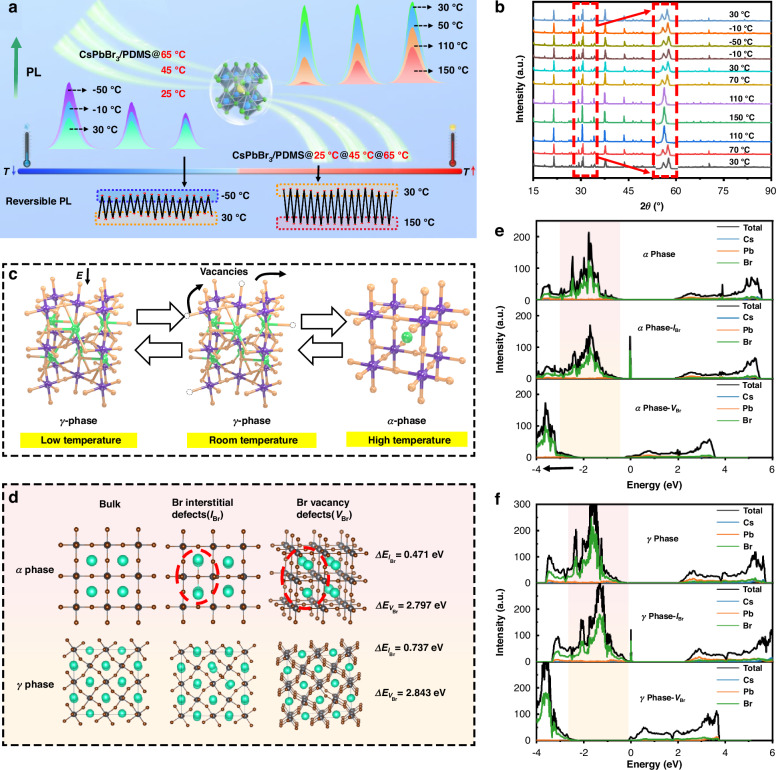
**Reversible response to temperature. a** Fluorescence changes of nanospheres with temperature. **b** XRD changes of nanospheres with temperature. **c** Crystal structure of *α*, *γ* phase. **d** Intrinsic defects and the corresponding defect formation energies. **e**, **f** DOS of *α*, *γ* phase and corresponding intrinsic defects

Our investigation, as depicted in Figs. 3a and S10, delves into the fluorescence responses of nanospheres at different temperature levels. As the temperature increases, the fluorescence of the gradient-stable nanospheres gradually decreases. Fluorescence attenuation of CsPbBr_3_/PDMS@25 °C nanospheres initiates at a lower temperature, whereas CsPbBr_3_/PDMS@65 °C nanospheres with superior stability begin to attenuate at a higher temperature. While fluorescence attenuation with increasing temperature is a common observation, we made a surprising discovery: as the temperature decreases, the fluorescence of the nanospheres starts to rise gradually. At lower freezing temperatures the CsPbBr_3_/PDMS@65 °C nanospheres, characterized by a higher shell crosslinking density, exhibit a more pronounced increase in fluorescence. Importantly, the fluorescence changes within these temperatures are also reversible for these nanospheres.

To comprehend the reversible fluorescence of CsPbBr_3_/PDMS nanospheres during heating and cooling, Fig. 3b illustrates the XRD patterns of CsPbBr_3_/PDMS@25 °C nanospheres at different temperatures. It is evident that as the temperature increases, the diffraction peaks near 30° begin to split, indicating the transition of CsPbBr_3_ from the *γ* phase to the *α* phase^36^. Conversely, when it drops to a warm temperature, CsPbBr_3_ reverts from the α phase to the *γ* phase. At frozen temperature, however, the diffraction peaks remain consistent with the *γ* phase (Fig. 3c).

Then, to investigate how the phase transition behavior affects the defect states and the subsequent reversible luminescence phenomenon, it is crucial to calculate the formation energies of intrinsic defects of CsPbBr_3_ in the *γ*, *α* phases and the corresponding density of states (DOS). Generally, the thermal defects consist of Br vacancy defects (*V*_Br_) and Br gap defects (*I*_Br_), also known as intrinsic defects, which serve as non-radiative recombination centers. Compared to the *V*_Br_ and *I*_Br_ defects in the α phase at high temperatures, the *γ* phase at warm/frozen temperatures exhibits higher defect-forming energy (Fig. 3d), hence the intrinsic *V*_Br_ and *I*_Br_ defects are energetically most preferred in the *α* phase. Heat induces vigorous motion and atomic rearrangement of CsPbBr_3_ from a dynamic standpoint, activating local defects and causing lattice distortion, thereby triggering phase transitions. However, the phase transition facilitates the return of dislocation ions to their original lattice, promotes self-elimination of defects during cooling, inhibits non-radiative recombination, and restores luminescence^37^.

The DOS of the *α* phase and *γ* phase exhibits similarities (Fig. 3e, f), with Br-4*p* and Pb-6*p* serving as the primary valence band maximums (VBM) and conduction band minimums (CBM)^38^. The backward shift in the Br state density indicates an increase in the electronic activity within Br, which is indicative of defects increase^37^. This may account for the quenching observed in CsPbBr_3_/PDMS nanospheres at high temperatures. Upon further cooling from a warm temperature, the frozen temperature may diminish defect activity by restricting the movement of unsaturated ions or altering the coordination environment, leading to defect passivation and fluorescence enhancement^39^. This is effectively supported by the TRPL curve in Fig. S11 and Table S2. When the temperature rises, the lattice energy is released, and the defects are restored. This process is similar to defect passivation and re-exposure.

High temperature may induce phase transitions and heat accumulation, resulting in the formation of non-radiative traps and the resulting strong thermal quenching of perovskites. However, in this work, high temperature does not readily lead to irreversible lattice degradation of CsPbBr_3_/PDMS nanospheres, due to the dual passivation and protection from DBSA ligands and PDMS shells^17,40^. Therefore, upon cooling, dislocation ions can rejoin the original lattice structure, effectively causing inherent defects and self-elimination of non-radiative recombination centers, thereby prompting fluorescence recovery. While further cooling to frozen temperature, the reduction in lattice system energy inhibits ion transfer and loss, promotes defects passivation, and boosts fluorescence intensity. Conversely, as temperature rises, lattice activity is reinstated, defects are exposed, and fluorescence decreases to its initial level.

#### Reversible response to pressure

**Fig. 4:**
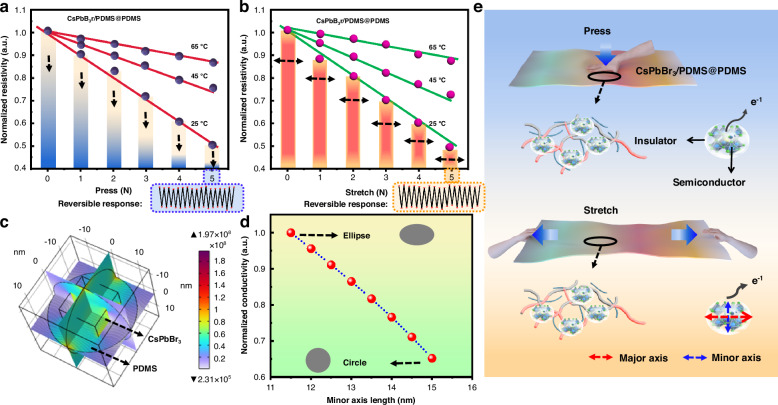
**Reversible response to pressure**. The electrical properties changes of CsPbBr_3_/PDMS@PDMS composite when being **a** pressed and **b** stretched. **c** Physical field simulation of the CsPbBr_3_/PDMS nanosphere. **d** The resistivity changes with the deformation of CsPbBr_3_/PDMS nanosphere. **e** Schematic diagram of the composite when being pressed and stretched

In addition to humidity and temperature, the pressure-sensitive properties of CsPbBr_3_/polymer nanospheres were discovered and analyzed thoroughly for the first time. When CsPbBr_3_/PDMS nanospheres were encapsulated within a flexible substrate PDMS, the electrical properties of the CsPbBr_3_/PDMS@PDMS composite material notably decreased when being pressed or stretched, with varying amplitudes of decrease observed for different nanospheres, as illustrated in Fig. 4a, b. The composite’s response to pressure is also found reversible. To elucidate the underlying reasons, we initially conducted the same mechanical strain tests on pure PDMS and CsPbBr_3_@PDMS composite, finding that the resistivity of both materials does not exhibit significant changes (Fig. S12). These results prove that the variations in resistivity stem from the PDMS shell we designed.

As illustrated in Figs. 4c and S13, through constructing the physical field simulation of CsPbBr_3_/PDMS nanosphere, it can be deduced that the alteration in resistivity may stem from deformation occurring when the composite experiences compression or stretching. Under pressure or tension, the polymer network within the material may deform (Fig. S14), causing concurrent deformation of the nanospheres. This deformation results in shortening along one direction, facilitating the escape of electrons from the internal semiconductor along the shorter axis (Fig. 4e)^41,42^. Figure 4d illustrates that as the PDMS shell transitions from spherical to ellipsoidal, the nanosphere’s resistivity linearly decreases. It is inferred that deformation may be the primary cause of the observed change in resistivity.

To further validate the crucial role of PDMS material characteristics, the shape of nanospheres, and even the dispersion of perovskite materials in pressure-sensitive properties, we also conducted pressure sensitivity tests on CsPbBr_3_/PMMA@PDMS and CsPbBr_3_/PS@PDMS composites (Fig. S15). The pressure-sensitive characteristics of the CsPbBr_3_/PMMA@PDMS composite exhibit irregularity with a small amplitude. Conversely, while the pressure-sensitive characteristics of the CsPbBr_3_/PS@PDMS composite demonstrate regularity, the changing extent is not pronounced. PS and PMMA possess high rigidity as plastic materials, resulting in less drastic amplitude changes upon deformation^43^. In contrast, PDMS, being a low-rigidity material, undergoes noticeable deformation under stretching or compression^44^, thus exhibiting relatively significant pressure-sensitive properties.

The disparity in the regularity governing pressure-sensitive characteristics may arise from the dispersion uniformity of PQDs within the nanosphere and the regularity of the nanosphere’s shape. We juxtaposed the morphologies of these three distinct perovskite/polymer composites on a uniform scale and further examined the encapsulation and dispersion of the CsPbBr_3_ PQDs from the perspective of corresponding high-resolution TEM (HRTEM) images with statistical data analysis (Fig. S15). The CsPbBr_3_/PDMS nanosphere is characterized by a uniform single-core single-shell morphology, with the crystal well wrapped in the middle of the nanosphere. In the case of the CsPbBr_3_/PS nanosphere, a regular multi-core single-shell structure is observed, where the CsPbBr_3_ PQDs are uniformly dispersed throughout the spherical PS matrix. In contrast, the CsPbBr_3_/PMMA composite presents an irregular core–shell structure. The CsPbBr_3_ PQDs exhibit size heterogeneity and their dispersion within the polymer is uneven, with some polymer regions containing only a few scattered CsPbBr_3_ PQDs.

The CsPbBr_3_ PQDs are adeptly encapsulated within the polymer matrix, a result of our innovative in-situ synthesis idea for perovskite/polymer composites, as shown in Fig. S16. The catalyst is anchored by CsPbBr_3_ PQDs through chemical bonding, facilitating the precise and exhaustive encapsulation through the integration of polymer monomer. This in-situ coating technique surpasses conventional physical blending methods by catering to surface modification requirements, ensuring comprehensive coverage of the PQDs, and circumventing issues of incomplete encapsulation.

The encapsulated polymer shell material acts as the primary channel for electron tunneling, where regular deformation significantly modulates the tunneling trajectory. A shell with a regular geometry offers a more precise and tunable electron tunneling pathway^45^. Furthermore, the uniform distribution of the sensing material throughout the nanospheres augments the consistency of the tunneling paths and their probabilities^46^. The CsPbBr_3_/PDMS and CsPbBr_3_/PS nanospheres exhibit regular morphologies with CsPbBr_3_ PQDs uniformly dispersed throughout. This uniform dispersion in the regular morphologies, in turn, endows these nanospheres with consistent pressure-sensitive properties (Fig. S15d, h). In contrast, the irregular shape of the CsPbBr_3_/PMMA composites, coupled with the non-uniform dispersion of the sensing material, exacerbates the irregularity of the pressure-sensitive properties (Fig. S15l).

Furthermore, we have also investigated the scenarios of partial embedding of CsPbBr_3_ PQDs within the polymer matrix and their distribution on the surface (Fig. S17a, b). This investigation offers novel insights into the utilization of perovskite/polymer composites for future pressure-sensitive applications. Simulation analyses reveal that alterations in conductivity still adhere to a linear progression. More importantly, as the embedded fraction of CsPbBr_3_ PQDs diminishes, the rate of conductivity decline attenuates (Fig. S17c). This observation may be attributed to variations in electron tunneling probabilities and pathways. As the tunneling barrier lessens, electron tunneling becomes more facile, thereby diminishing the impact on the decline in conductivity variations^47^. Thus, the important role of encapsulated shell materials in electron tunneling is further verified.

### Applications on multi-dimensional sensing and interactive displays

The sensitive and reversible responses of CsPbBr_3_/PDMS nanospheres offer fresh insights into the realm of multi-dimensional sensing and interaction. These nanospheres, possessing gradient photoelectrical properties and stability, exhibit distinctive behaviors under varying stimuli, highlighting the specificity crucial for perception. Such multifunctional CsPbBr_3_/PDMS nanospheres have the potential to serve as foundational materials for smart sensing or interactive devices.

**Fig. 5:**
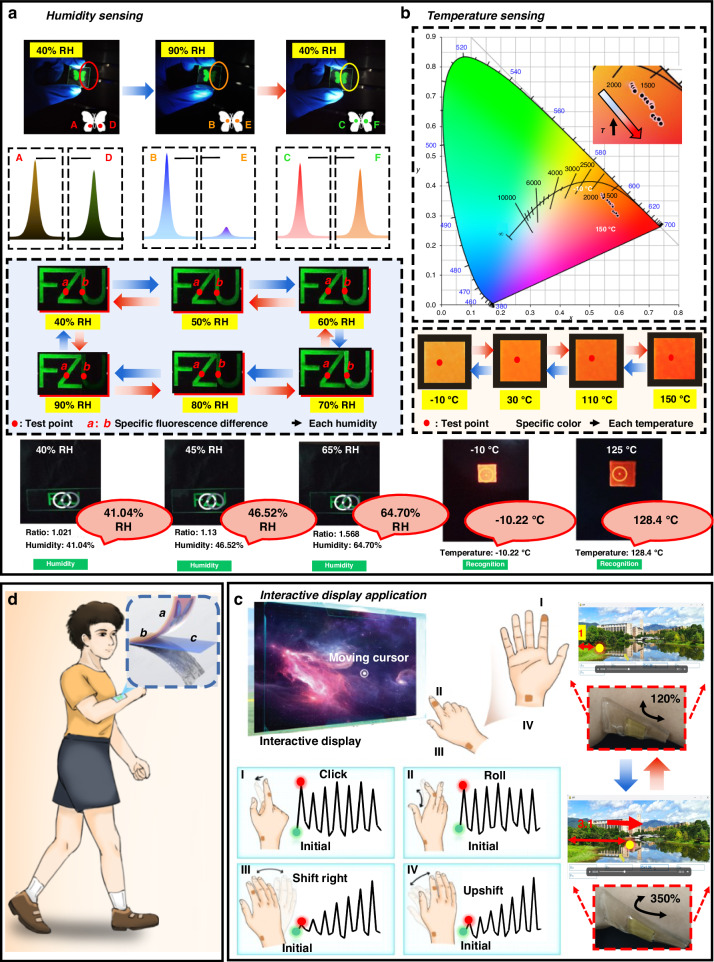
**Multi-dimensional sensing and interactive displays. a** Humidity sensing. **b** Temperature sensing. **c** Interactive displays application. **d** Conceptual diagram of CsPbBr_3_/PDMS nanospheres applications

Figure 5a validates the humidity sensing capability of CsPbBr_3_/PDMS nanospheres. By utilizing CsPbBr_3_/PDMS@25 °C and CsPbBr_3_/PDMS@65 °C nanospheres for pattern printing, distinct responses are observed under varying humidity levels. Under normal conditions (40% RH), both nanospheres emit bright green light, forming a clear butterfly shape. However, at high humidity levels (90% RH), the fluorescence of the CsPbBr_3_/PDMS@25 °C nanospheres filled region is quenched, while that of the CsPbBr_3_/PDMS@65 °C nanospheres filled region intensifies, albeit forming only half of the butterfly shape. Upon returning to 40% RH, the butterfly shape reemerges. This reversible modulation of fluorescence enables humidity perception and also serves as an anti-counterfeiting measure.

In addition to distinguishing between high and low humidity, environmental humidity perception also involves evaluating intermediate humidity states. This capability addresses a limitation in human skin’s ability to perceive and differentiate such nuanced humidity levels. Similarly, the substrate includes the inscription “FZU”, where “FZ” filled CsPbBr_3_/PDMS@65 °C nanospheres and “U” filled CsPbBr_3_/PDMS@25 °C nanospheres. From 40% RH to 70% RH, the fluorescence of CsPbBr_3_/PDMS@25 °C nanospheres exhibits a linear increase, followed by a decline. However, relying solely on CsPbBr_3_/PDMS@25 °C nanospheres, the fluorescence between 70% and 80% RH may resemble that between 40% and 70% RH. Thus, CsPbBr_3_/PDMS@65 °C nanospheres in the “FZ” region aid in judgment coordination. This is because the fluorescence of CsPbBr_3_/PDMS@65 °C nanospheres is amplified at 70% to 80% RH while remaining temporarily stable at 40–70% RH. Consequently, the specific fluorescence difference of “FZ” and “U” can represent each humidity level to achieve perceptual function (corresponding to test points “*a*” and “*b*” in Fig. 5a).

Figure 5b validates the temperature sensing application of CsPbBr_3_/PDMS nanospheres. Utilizing nanospheres with gradient stability can expand the range of temperature measurement and fluorescence variation. Specifically, when relying solely on CsPbBr_3_/PDMS@25 °C nanospheres for temperature determination, significant fluorescence variations may not be observed at frozen or higher temperatures. On the other hand, using only CsPbBr_3_/PDMS@65 °C nanospheres for judgment may result in minimal fluorescence changes below 100 °C, rendering temperature determination via fluorescence difficult at lower warm temperatures.

To address these challenges, a combination of three types of nanospheres is employed to broaden the temperature measurement range and fluorescence variations. These nanospheres, along with stable Cd-based red QDs, act as raw materials for temperature determination. The addition of red QDs serves two main purposes: altering the color to a more noticeable orange-red hue and helping differentiate it from the aforementioned humidity-sensing color, which is adjusted to warm tones. This integration of functionalities allows for clear differentiation between temperature and humidity judgments. Temperature judgment can be inferred by observing the color change from (0.5413, 0.3718) to (0.5950, 0.3000). It is worth mentioning that at temperatures higher than 150 °C, the fluorescence reversible recovery ability deteriorates due to irreversible fluorescence quenching in some PQDs (Fig. S18).

Dedicated to improving the convenience and accuracy of perception and interaction, we have developed a predictive model that combines the brightness data from humidity sensing and the color data from temperature sensing. This model, integrated into a user-friendly mobile application (APP), enables users to achieve over 95% accuracy in perceiving humidity and temperature levels through camera scanning (refer to the bottom of Fig. 5a, b). In addition to this, looking to the future, we can also integrate the cutting-edge technology of nanowire spectrometers on the perception diaphragm^48^. The introduction of the nanowire spectrometer will undoubtedly be an important solution to improve the convenience of integrated systems and give our wearable devices unprecedented monitoring accuracy and analysis depth (Fig. S19).

Moreover, these nanospheres with pressure sensitivity allow for their integration into a unified unit capable of sensing humidity, temperature, and pressure simultaneously. The pressure sensing layer can be strategically positioned at the base (Fig. 5d). Figure 5c illustrates the practical application of pressure sensing with CsPbBr_3_/PDMS@25 °C nanospheres, which exhibit the most significant electrical performance variation.

The CsPbBr_3_/PDMS@PDMS composite is attached to different hand joints, enabling joint movements to induce variations in microcurrent. These changes interact with the display device through gesture controls that mimic a mouse’s functionality. For instance, positioning the composite at location “I” enables “click” interaction with the display device, while “II” facilitates “scroll” interaction. Similarly, placement at position “III” allows for “left and right movement” interaction, and at position “IV” for “up and down movement” interaction.

To validate the efficacy of these interactive functions, we have designed an interactive algorithm that demonstrates controlling the screen cursor’s left and right movement via gestures. Our observation reveals that the proportional changes in gesture arc closely mirror the changes in mouse movement on the display screen, achieving an interaction accuracy of approximately 95% (where the composite stretched 2.9 times longer, while the cursor moved 3.1 times more on the screen).

## Discussion

In this study, inspired by the neurons, we propose a versatile sensing material design comprising CsPbBr_3_/PDMS nanospheres, capable of simultaneously detecting and responding to humidity, temperature, and pressure. PDMS serves a dual purpose: firstly, it shields the internal perovskite material, preserving its reversible activity. Secondly, leveraging its porous characteristics, PDMS facilitates direct contact between CsPbBr_3_ and the environment, enhancing the material’s sensitivity. Notably, the tunable crosslinking density of PDMS allows for the creation of nanospheres with customized sensitivity and reversibility to different levels of stimuli. This variation in response can be likened to the weighting characteristics observed on dendrites, providing unique responsiveness and perception capabilities.

Importantly, we delve into the mechanisms governing the differentiated sensitivity and reversibility of CsPbBr_3_/PDMS nanospheres to different stimuli. Humidity response relies on the degradation and recrystallization of the crystal lattice, coupled with reversible surface defect passivation. Temperature sensitivity and invertibility are linked to the reversible phase transition and energy fluctuations within the system. Pressure response mainly stems from the regular reversible deformation of the low-rigidity PDMS shell, along with the uniform distribution of the CsPbBr_3_ core material. Ultimately, the integrated receptor based on CsPbBr_3_/PDMS nanospheres not only enables accurate humidity and temperature detection but also shows promise in human–computer interaction, with perception and interaction accuracy exceeding 95%. This intriguing discovery opens up exciting possibilities for advancements in the realms of multi-dimensional sensing and interactive display applications.

## Materials and methods

### Materials

Cesium carbonate (Cs_2_CO_3_, Sigma-Aldrich, 99.9%), Octadecene (ODE, Sigma-Aldrich, 90%), Oleylamine (OAm, Aladdin, 80–90%), Lead (II) bromide (PbBr_2_, Aladdin, 99%), Dodecylbenzenesulphonic acid (DBSA, Aladdin, 90%), Octamethylcyclotetrasiloxane (D4, Aladdin, 98%), Toluene (C_6_H_5_CH_3_, Sigma-Aldrich, AR).

### Synthesis of DBSA-CsPbBr_3_ PQDs

Firstly, CsCO_3_ (0.203 g), ODE (10 mL), and DBSA (1 mL) were mixed in a three-necked flask under a nitrogen atmosphere. The mixture underwent vigorous magnetic stirring for 1 h at 120 °C until the solid in the solution completely dissolved. Subsequently, PbBr_2_ (0.0745 g) and ODE (12 mL) were added to another three-necked flask, and nitrogen was passed into the flask at 80 °C for 1 h. The temperature was then raised to 120 °C and then DBSA (0.5 mL) and OAm (1.5 mL) were added. When PbBr_2_ completely dissolved, the temperature was raised to 180 °C, and 1 mL cesium precursor was quickly added. After reacting for 10 s, the liquid was cooled in a water bath. After cooling to room temperature, the reaction mixture was poured into a dry centrifuge tube and centrifuged several times at 6000 rpm for 8 min. The supernatant, after centrifugation, was re-dispersed in toluene and stored for later use.

### Synthesis of CsPbBr_3_/PDMS nanospheres

D4 (1.5 mL) was added to the DBSA-CsPbBr_3_ (5 mL) obtained in the above steps and stirred at 65 °C and 500 rmp magnetic stirring for 12 h. The viscous solution obtained was stored for further use.

### Characterization

The transmission electron microscope (TEM) observation was performed with a FEI TECNAI G2 F20 (FEI Corporation). The PL spectra were tested by UV-3600 (Shimadzu). TRPL was collected by FLS980 (Edinburgh). XRD patterns were collected by using DY1602/Empyrean (Panaco). Ultrafast TA was collected by Helios (Ultrafast systems). Viscosity was collected by Brookfield DV-2 pro (Brookfield). Resistivity was collected by ST2258C (Suzhou Lattice Electronics Co., Ltd). Color coordinates were measured by SRC-200M (Hangzhou Everfine Photo-E-Info Co., Ltd).

### Calculation and simulation details

We performed the first-principles calculations in the frame of DFT with the program package CASTEP, using the plane-wave ultra-soft pseudopotential (PW-USPP) method and the Perdew–Burke–Ernzerhof (PBE) form of generalized-gradient approximation (GGA) exchange-correlation energy functional. The structure optimization of CsPbBr_3_ has been carried out using means of the Broyden–Fletcher–Goldfarb–Shanno (BFGS) algorithm by allowing all atomic positions to vary and relaxing lattice parameters. They would stop until the total energies converged to 10^−5^ eV atom^−1^, the forces on each unconstrained atom were smaller than 0.03 eV Å^−1^, the stresses were lower than 0.05 GPa, and the displacements were <0.001 Å. The plane-wave cutoff, Ecut, was chosen to be 340 eV. The *k*-point mesh of 4 × 4 × 4 was used for Brillouin zone (BZ) sampling for FAPbI_3_. Physical field simulations were conducted using the AC/DC module of multiphysics simulation software to study the behavior of the structure under various conditions. The entire structure was meshed by a physics-controlled method, taking into account the differing relative permittivity properties of the perovskite and PDMS materials. Bias and ground connections were applied to the device to obtain the steady-state current distribution. To discuss the water molecule transport behaviors, the molecular dynamics durations were set as 500-ps to get more information. We performed a molecular dynamics simulation under 298 K to determine how the structure was affected. The Nose method was adopted to maintain the assigned pressure, in which the *Q* ratio was set as 0.01. The non-equilibrium molecular dynamics method performed with the Peral script was used to calculate the thermal conductivity of the amorphous systems. In this study, the script for calculating the thermal conductivity has been written based on the imposed flux method of Jund^49^. The thermal conductivity was predicted by giving the user input parameters such as, xsd file name, force field, and thermostat.

## Supplementary information

Supplementary Information for: Neuron-inspired CsPbBr3/PDMS Nanospheres for Multi-dimensional Sensing and Interactive Displays

## Data Availability

The data that support the findings of this study are available from the corresponding author upon reasonable request.
