# Supplementary material for: Neuron-inspired CsPbBr3/PDMS nanospheres for multi-dimensional sensing and interactive displays
Source: Light Sci Appl. 2025 Jan 17;14:55. doi: 10.1038/s41377-025-01742-z (PMC11742394; doi:10.1038/s41377-025-01742-z)
Supplement: Supplementary file 1 — Supplementary Information for: Neuron-inspired CsPbBr3/PDMS Nanospheres for Multi-dimensional Sensing and Interactive Displays [file 41377_2025_1742_MOESM1_ESM.doc]

## Supplementary Information for:

**Neuron-inspired CsPbBr_3_/PDMS Nanospheres for Multi-dimensional** **Sensing and Interactive Displays**

Junhu Cai^1^, Xiang Zhang^,3^, Yu Chen^1^, Wenzong Lai^1^, Yun Ye^1,2^, Sheng Xu^1,2^, Qun Yan^1,2^, Tailiang Guo^1,2^ , Jiajun Luo^3^, and Enguo Chen^1,2*^

^1^National and Local United Engineering Laboratory of Flat Panel Display Technology, College of Physics and Information Engineering, Fuzhou University, Fuzhou 350108, China.

^2^Fujian Science & Technology Innovation Laboratory for Optoelectronic Information of China, Fuzhou 350108, China.

^3^Wuhan National Laboratory for Optoelectronics and School of Optical and Electronic Information, Huazhong University of Science and Technology, Wuhan 430074, China.

***Correspondence:** Enguo Chen (ceg@fzu.edu.cn)

**Section S1.**


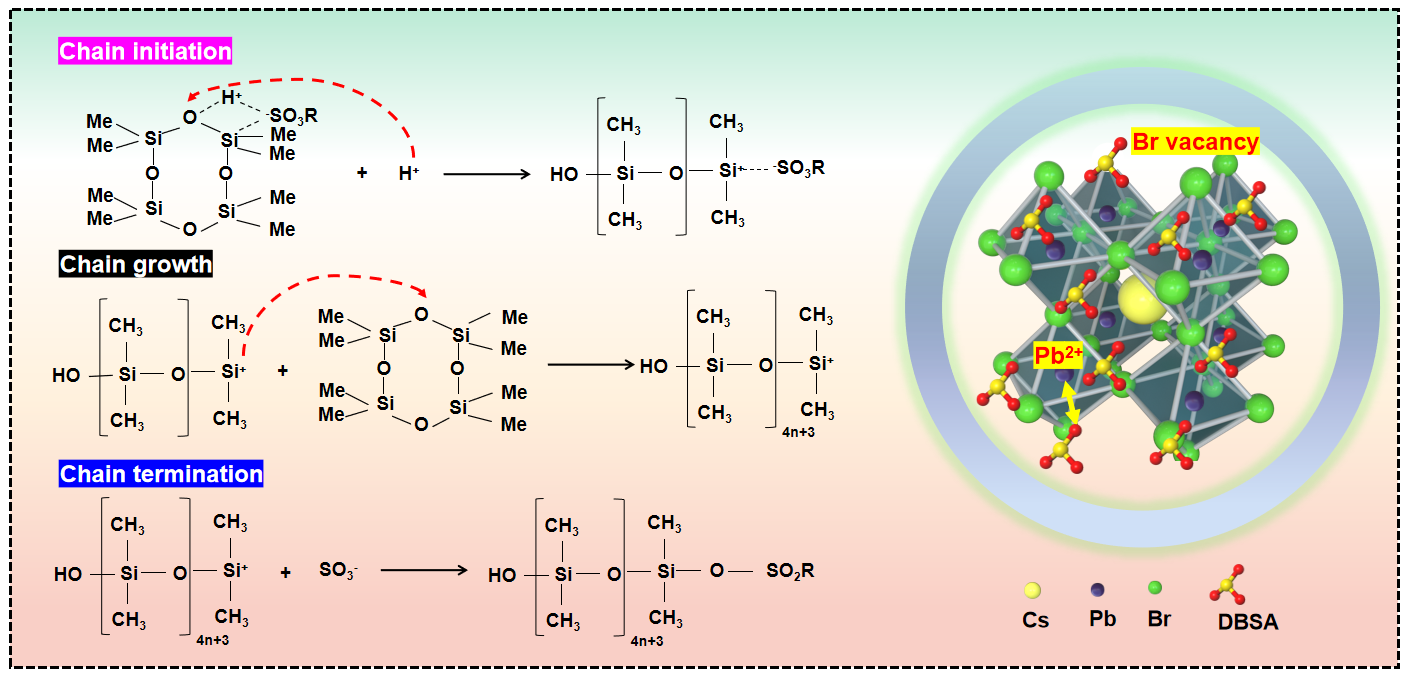


**Fig. S1** CsPbBr_3_/PDMS nanosphere polymerization principle, including chain initiation, chain growth and chain termination.

**Section S2.**


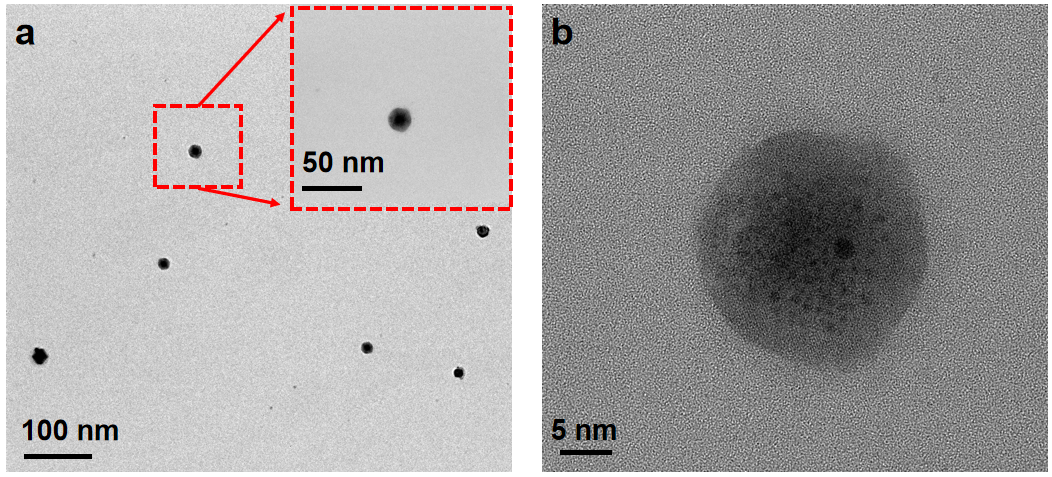


**Fig. S2** **a** TEM and **b** HRTEM images of CsPbBr_3_/PDMS nanospheres.

**Section S3.**


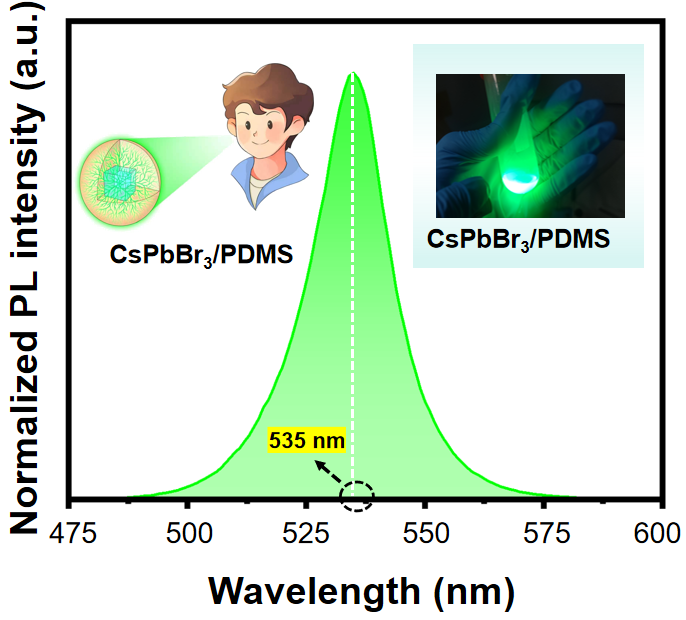


**Fig. S3** PL spectra of CsPbBr_3_/PDMS nanospheres.

**Section S4.**


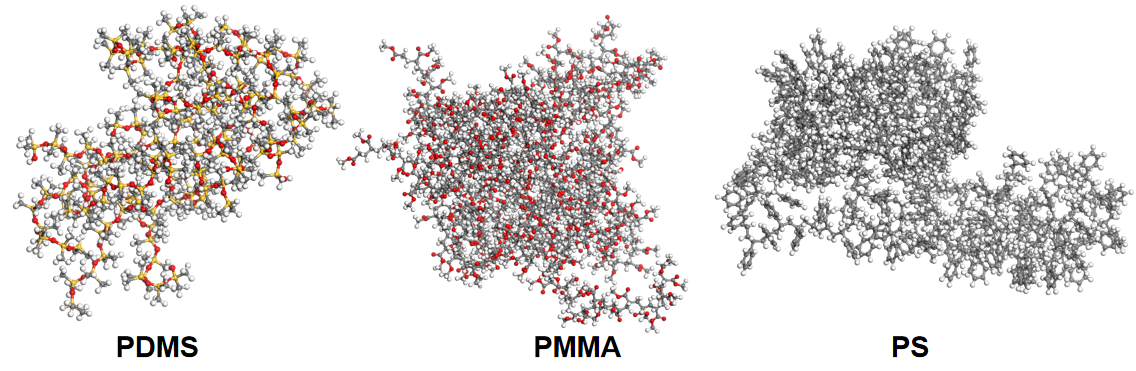


**Fig. S4** Model diagrams of PDMS, PS and PMMA.

**Section S5.**


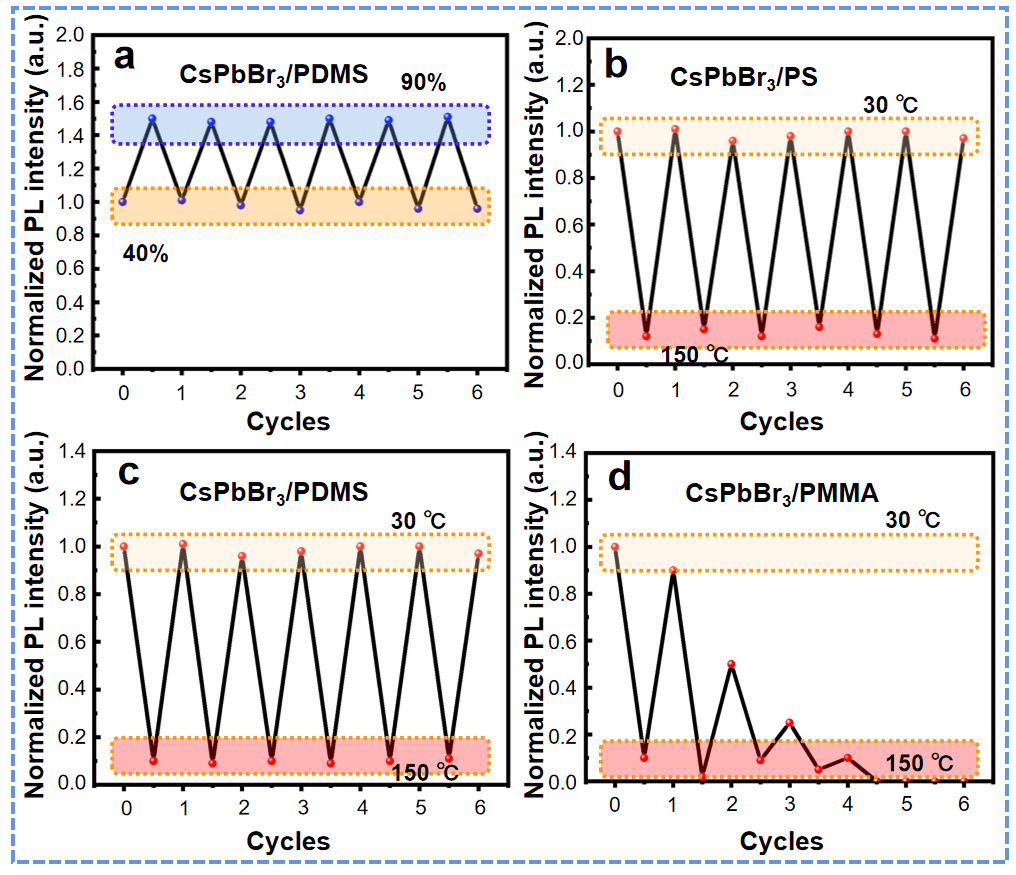


**Fig. S5** **a** Reversible response of CsPbBr_3_/PDMS to humidity (40% RH and 90% RH). Reversible response of **b** CsPbBr_3_/PS **c** CsPbBr_3_/PDMS and **d** CsPbBr_3_/PMMA to temperature (30 ℃ and 150 ℃).

**Section S6.**


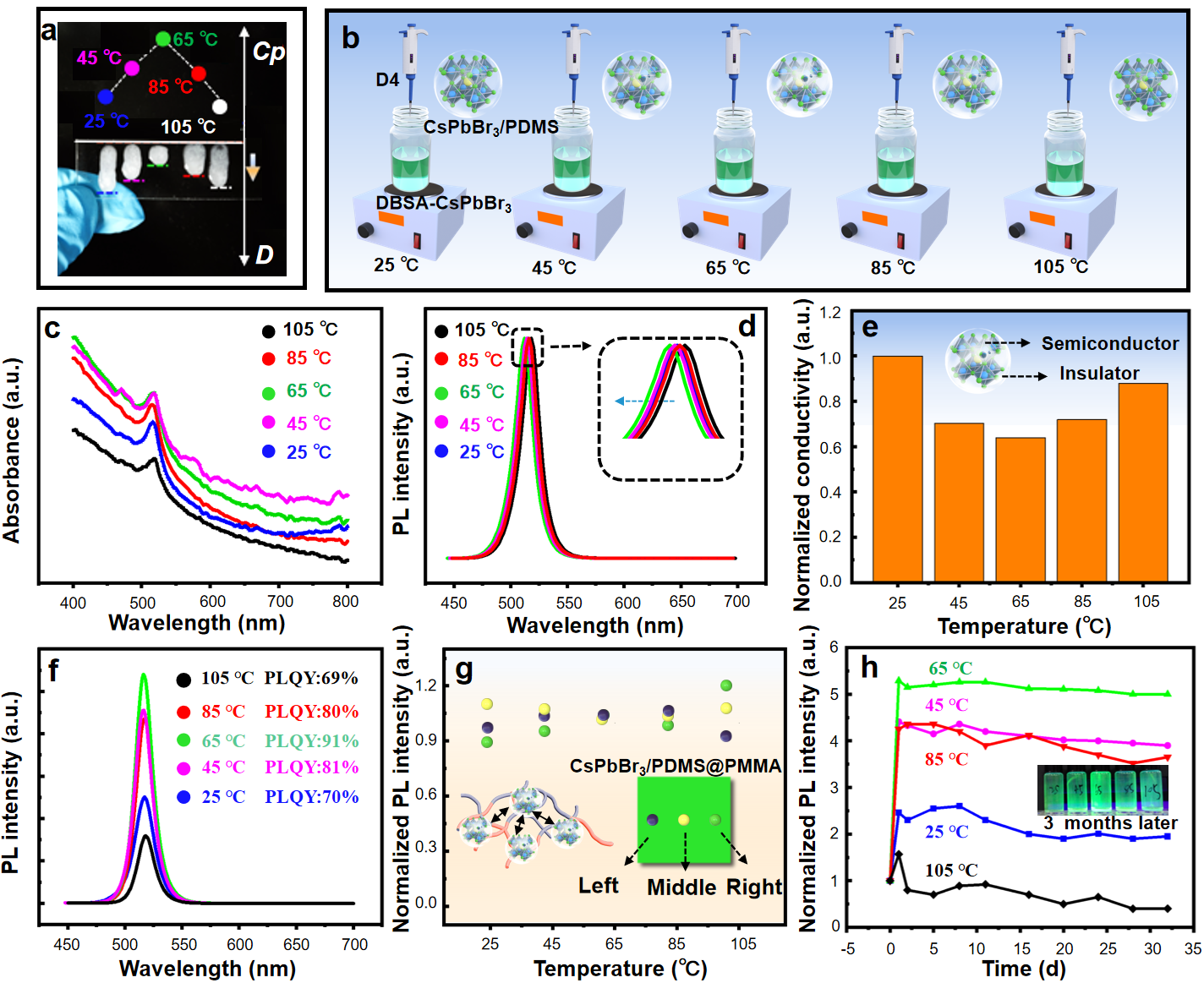


**Fig. S6** **a** PDMS obtained by polymerization of D4 at gradient temperatures (25 ℃, 45 ℃, 65 ℃, 85 ℃ and 105 ℃). **b** CsPbBr_3_/PDMS nanospheres prepared at gradient polymerization temperature. **c** UV-vis absorption, **d** PL spectra, **e** resistivity, **f** PLQY, **g** PL uniformity and **h** PL stability of CsPbBr_3_/PDMS nanospheres with gradient photoelectrical properties.

By setting the polymerization temperature of 25 ℃, 45 ℃, 65 ℃, 85 ℃ and 105 ℃, we found that the MW of D4 products catalyzed by DBSA did increase first and then decrease, and the MW of products was the highest at 65 ℃. Since the MW is proportional to the viscosity^1^ (**Fig. S6a**). The coating of PDMS with controllable MW may bring new properties to the CsPbBr_3_/PDMS nanospheres. We added D4 to the synthesized DBSA-CsPbBr_3_ and then the nanospheres were prepared by the same gradient polymerization temperature (25 ℃, 45 ℃, 65 ℃, 85 ℃ and 105 ℃) (**Fig. S6b**). Through a series of investigations, we found that the photoelectric properties and stability of the CsPbBr_3_/PDMS nanospheres would change with the MW of the PDMS shell, and the same trend would appear. We arrive at this view through the following test representations: (1) As shown in **Fig. S6c** and **d**, with the increase of polymerization temperature, the positions of the first exciton absorption peak and PL peak first shifted blue and then red-shifted. The cause may be attributed to the lattice contraction and the quantum size effect^2^. The larger the MW of the PDMS shell may be the greater the confined on the CsPbBr_3_ PQDs. (2) **Fig. S6e**, The change of the resistivity of the CsPbBr_3_/PDMS nanospheres is the same as that of the MW of PDMS, which may be due to the stronger binding ability of insulating organic compound (PDMS) with greater MW on the electron escape of perovskite semiconductor (CsPbBr_3_). (3) **Fig. S6f** shows the PLQY of the nanospheres. The PLQY of CsPbBr_3_/PDMS nanospheres prepared at 65 ℃ was the highest, reaching 92%. To some extent, MW is proportional to density. The higher the MW of PDMS and the higher coating density, the better the passivation effect of Si-O bond on CsPbBr_3_ PQDs^3^. (4) The PL uniformity of CsPbBr_3_/PDMS/PMMA film was characterized in **Fig. S6g**. The higher the MW of PDMS coating, the higher the surface viscosity of CsPbBr_3_/PDMS nanospheres. When the nanospheres are dispersed in PMMA, the contraction rate of the nanospheres is slow and cannot reach the critical value due to the limiting effect of the polymer with high surface viscosity. At this time, the normal stress of the nanospheres counteracts the capillary force driving the movement of the contact line, and the rebound of the nanospheres is completely inhibited, and the flow and dispersion of the nanospheres are uniform in the polymer matrix^4^. Therefore, the PL uniformity of CsPbBr_3_/PDMS@65 ℃/PMMA film is the best. (5) Given that the greater the MW, the denser the polymer network, the degree of protection for CsPbBr_3_ NCs will also change with the MW (**Fig. S6h**). The stability of CsPbBr_3_/PDMS nanospheres at 65 ℃ is the best. It is worth mentioning that although the MW at 105 ℃ polymerization temperature is larger than that at 25 ℃ polymerization temperature, long-term high-temperature polymerization will also destroy the CsPbBr_3_ itself, so the CsPbBr_3_/PDMS@105 ℃ nanospheres are less stable than the CsPbBr_3_/PDMS@25 ℃ nanospheres.

**Section S7.**


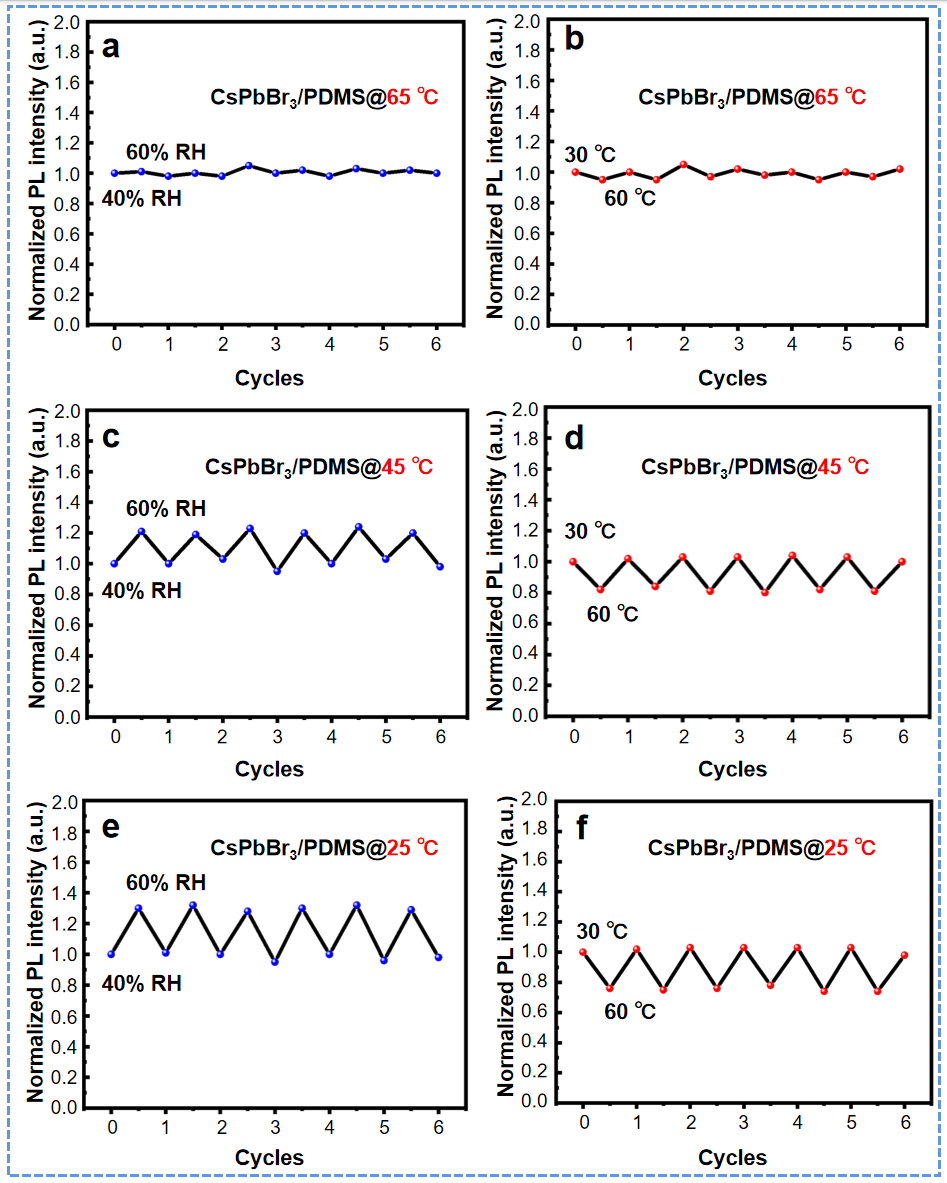


**Fig. S7** Reversible response of CsPbBr_3_/PDMS@65 ℃ to **a** humidity (40%RH and 60% RH) and **b** temperature (30 ℃ and 60 ℃). Reversible response of CsPbBr_3_/PDMS@45 ℃ to **c** humidity (40% RH and 60% RH) and **d** temperature (30 ℃ and 60 ℃). Reversible response of CsPbBr_3_/PDMS@25 ℃ to **e** humidity (40% RH and 60% RH) and **f** temperature (30 ℃ and 60 ℃).

**Section S8.**


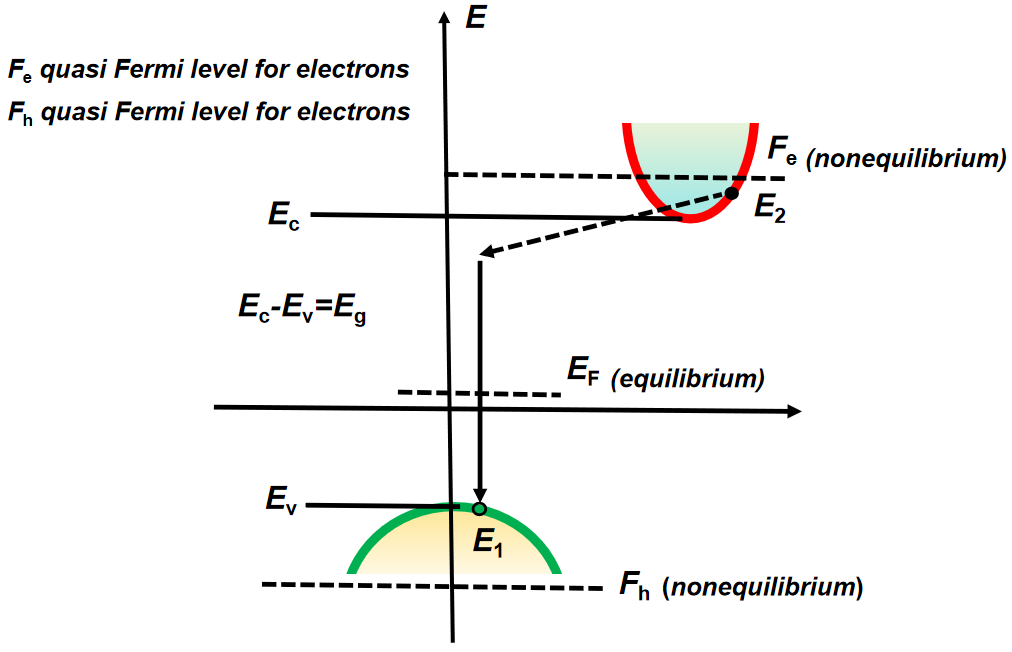


**Fig. S8** Schematic drawing of band structure for indirect semiconductors.

**Section S9.**


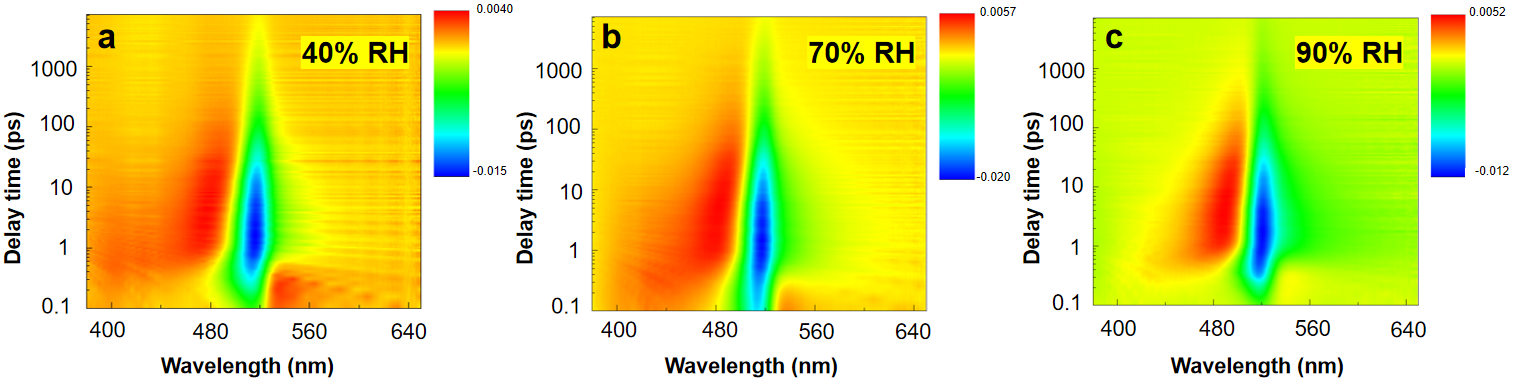


**Fig. S9** TA comparisons of nanospheres with humidity of **a** 40% RH, **b** 70% RH and **c** 90% RH.

**Section S10.**


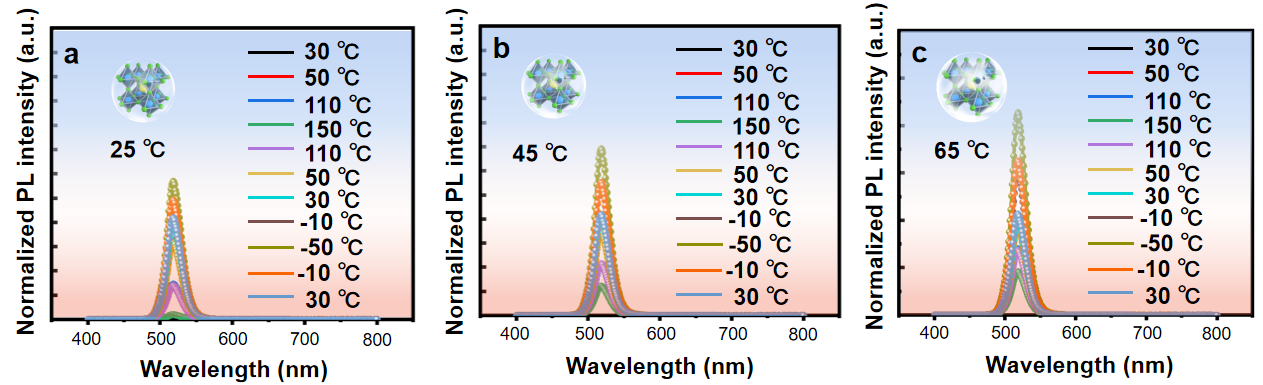


**Fig. S10** Fluorescence change curves of **a** CsPbBr_3_/PDMS@25 ℃, **b** CsPbBr_3_/PDMS@45 ℃ and **c** CsPbBr_3_/PDMS@65 ℃ nanospheres during the heating-cooling cycle.

**Section S11.**


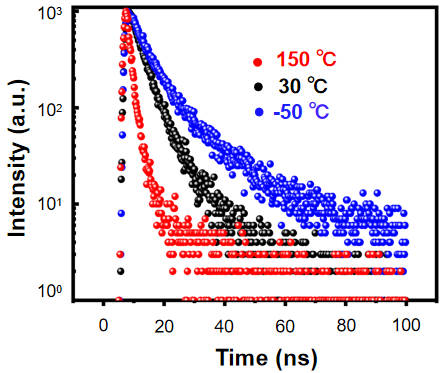


**Fig. S11** Variable temperatures TRPL curves of CsPbBr_3_/PDMS@25 ℃ nanospheres.

**Section S12.**


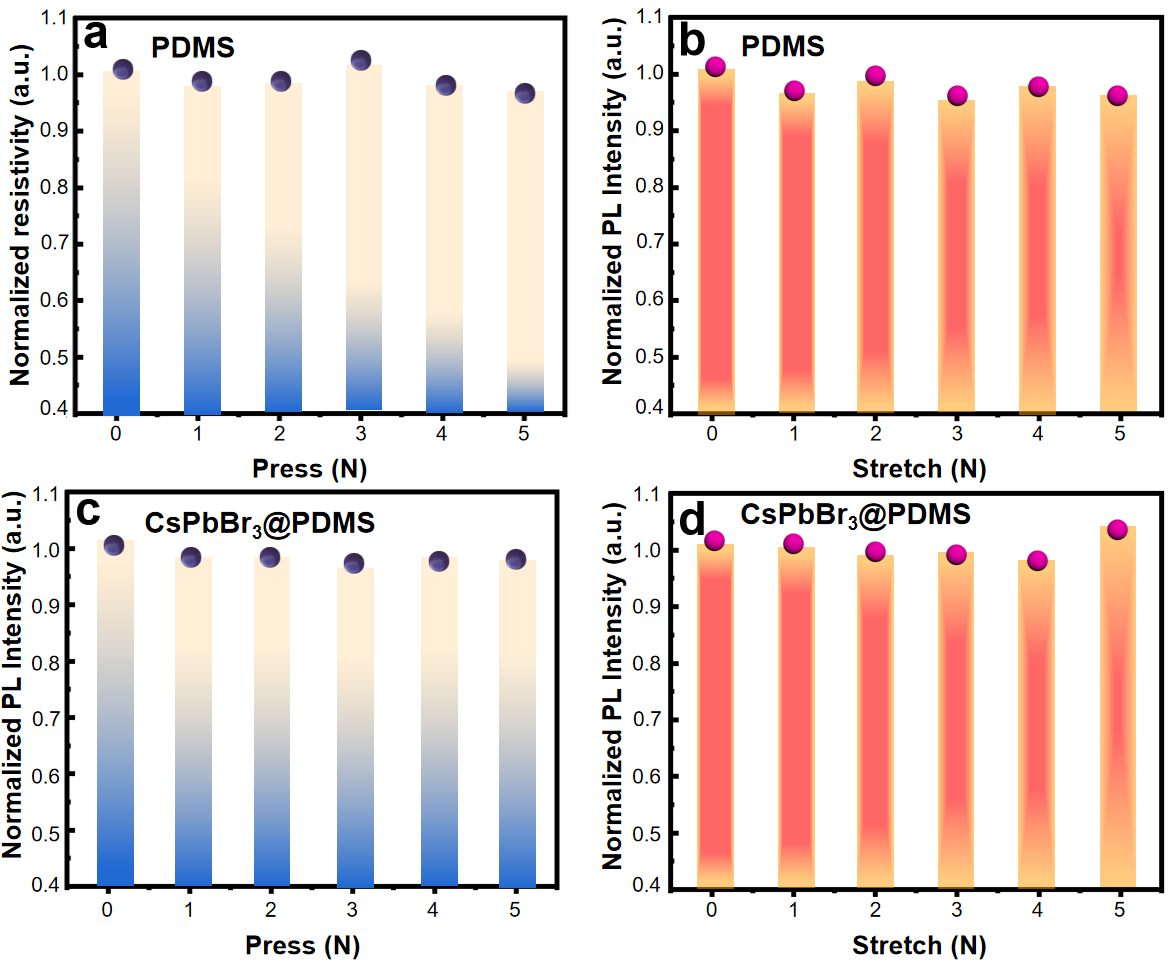


**Fig. S12** The resistivity changes of **a** PDMS and **b** CsPbBr_3_@PDMS composite when being pressed and stretched.

**Section S13.**


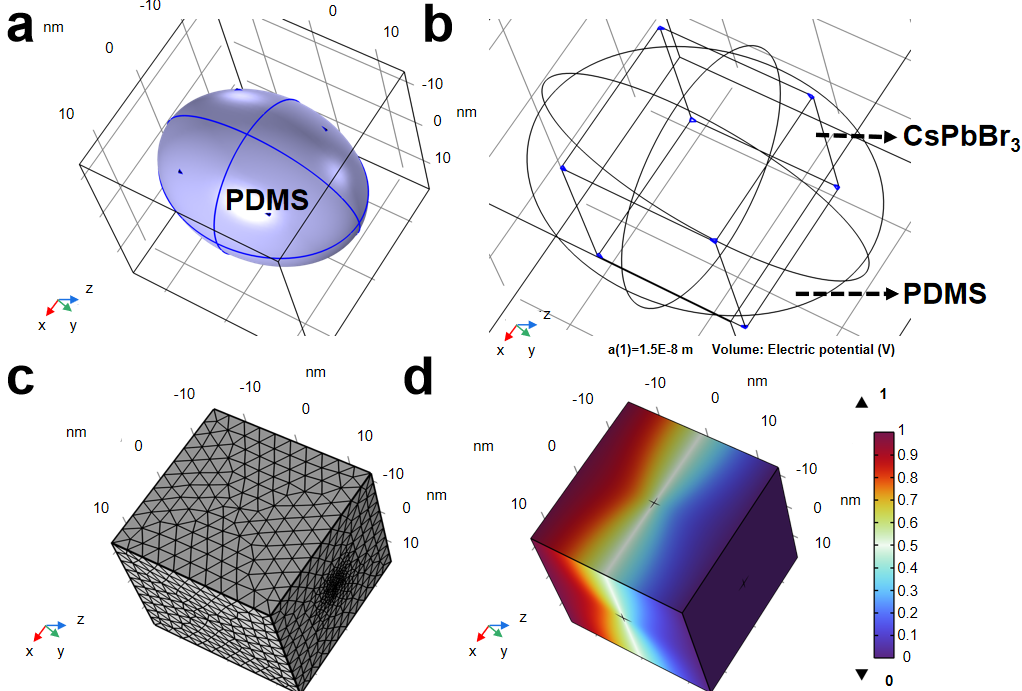


**Fig. S13** **a**-**d** Details of physical field simulation for the CsPbBr_3_/PDMS nanosphere.

**Section S14.**


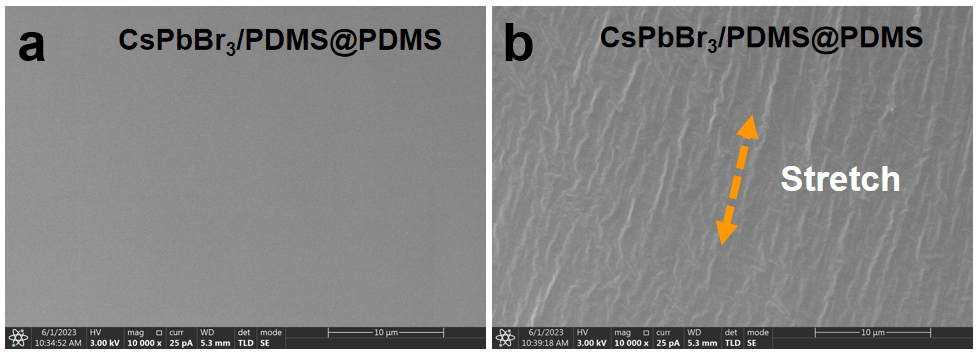


**Fig. S14** Surface morphology of the CsPbBr_3_/PDMS@PDMS **a** before and **b** after stretching.

**Section S15.**


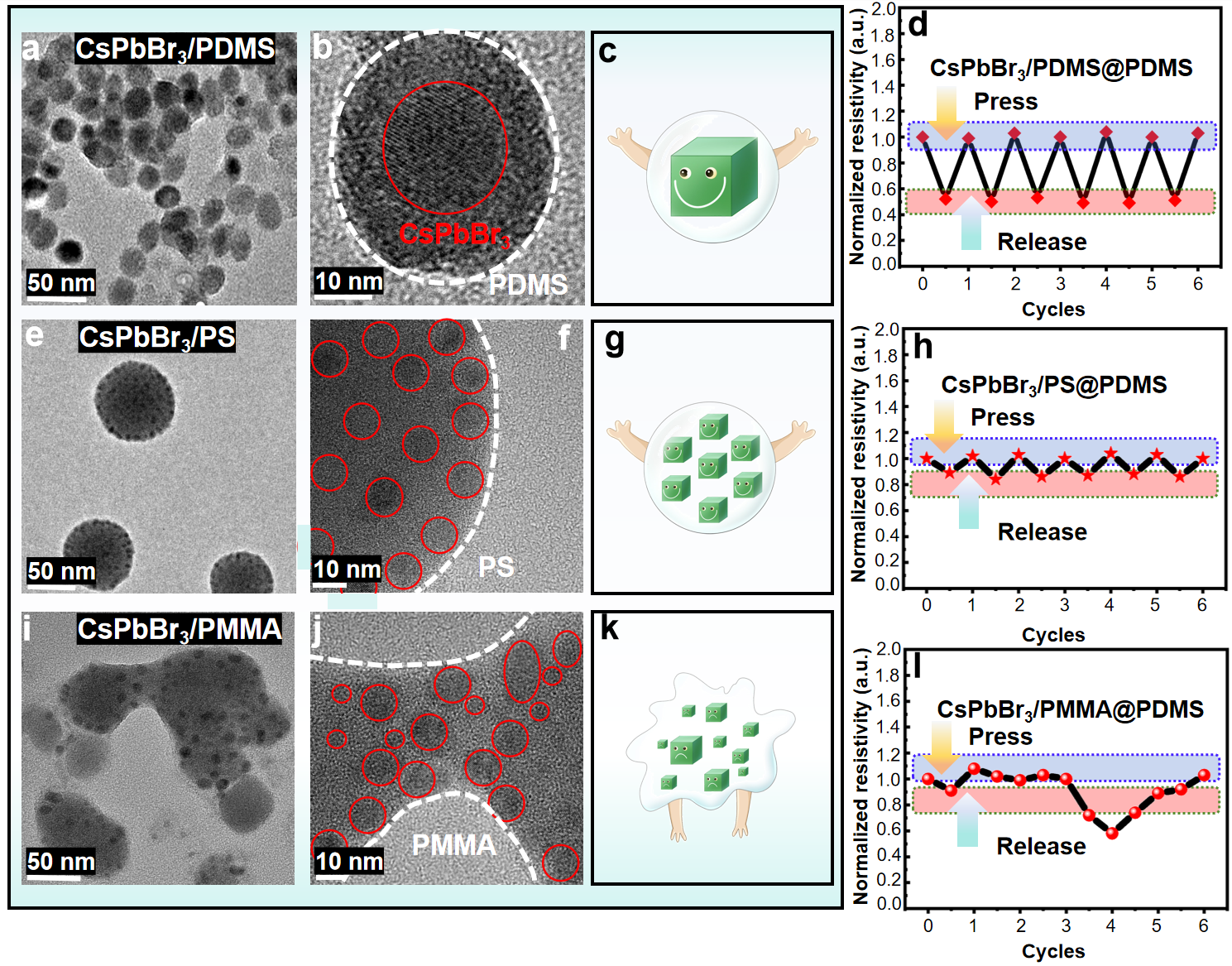


**Fig. S15** TEM, HRTEM, and schematic diagram of **a-c** CsPbBr_3_/PDMS nanospheres, **e-g** CsPbBr_3_/PS nanospheres and **i-k** CsPbBr_3_/PMMA composites. Pressure-sensitive characteristics of **d** CsPbBr_3_/PDMS@PDMS, **h** CsPbBr_3_/PS@PDMS, and **l** CsPbBr_3_/PMMA@PDMS composites.

**Section S16.**


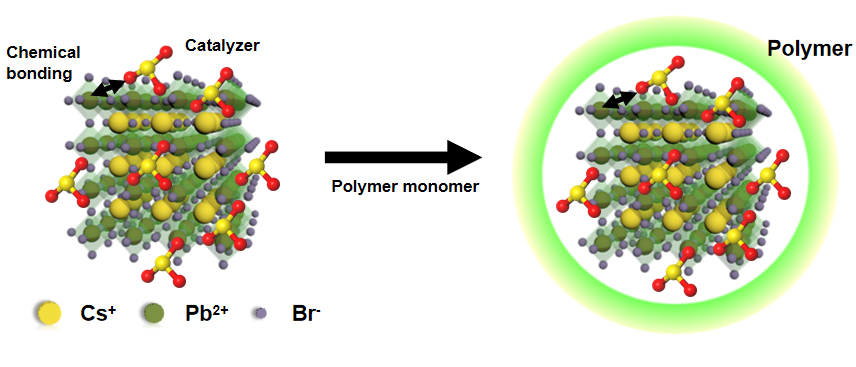


**Fig. S16** In situ synthesis idea for perovskite/polymer composite.

**Section S17.**


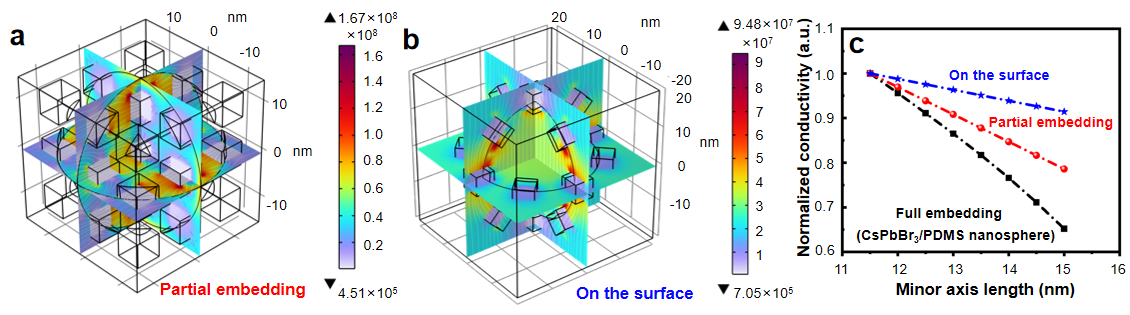


**Fig. S17** Physical field simulation of the scenarios for **a** partial embedding of CsPbBr_3_ PQDs within the polymer matrix and **b** their distribution on the polymer surface. **c** The conductivity changes with the deformation of CsPbBr_3_/PDMS nanosphere.

**Section S18.**


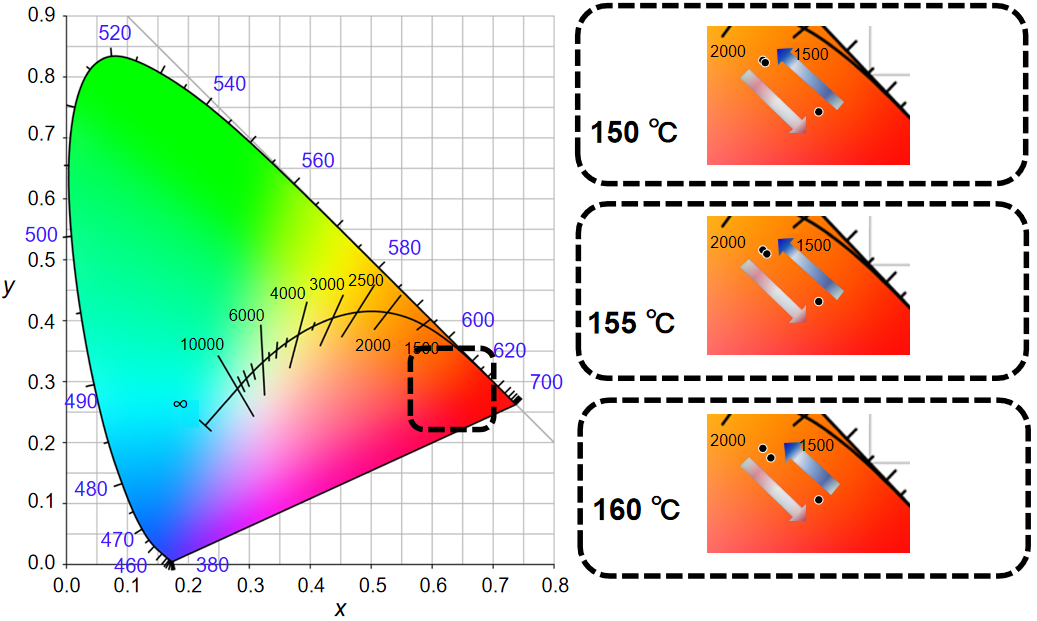


**Fig. S18** Fluorescence cooling recovery of the temperature measuring diaphragm at 150, 155 and 160 ℃.

**Section S19.**


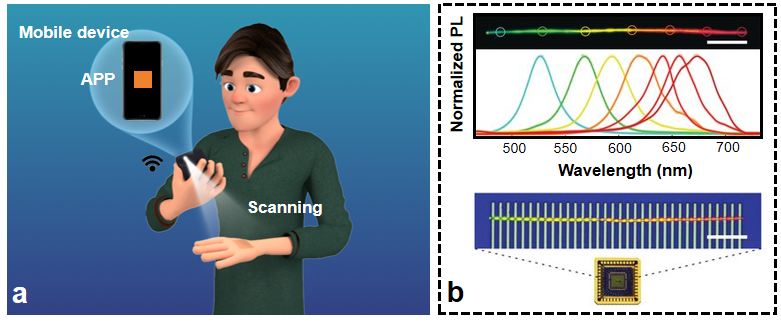


**Fig. S19 a** Schematic diagram of interaction through mobile devices. **b** A typical nanowire spectrometer^5^.

**Section S20.**

Table 1. TRPL data for CsPbBr_3_/PDMS under different RH.

| Fitting parameter | 40 RH | 70 RH | 90 RH |
| --- | --- | --- | --- |
| A_1_ | 2709.8 | 2847.3 | 2315.5 |
| τ_1_ (ns) | 32.3 | 31.2 | 34.2 |
| A_2_ | 336.1 | 452.2 | 258.4 |
| τ_2_ (ns) | 62.2 | 75.4 | 31.1 |
| τ_avg_ (ns) | 36.5 | 43.7 | 32.3 |

Table 2. TRPL data for CsPbBr_3_/PDMS@25 ℃ at different temperature.

| Fitting parameter | -50 ℃ | 30 ℃ | 150 ℃ |
| --- | --- | --- | --- |
| A_1_ | 801.0 | 723.3 | 682.2 |
| τ_1_ (ns) | 5.2 | 6.1 | 9.2 |
| A_2_ | 123.5 | 111.3 | 101.5 |
| τ_2_ (ns) | 28.1 | 22.2 | 10.4 |
| τ_avg_ (ns) | 16.2 | 12.7 | 9.2 |

**References**

1. Zhao, L. et al. Using viscosity modifying admixture to reduce diffusion in cement-based materials: Effect of molecular mass. *Construction and Building Materials* **290**, 123207 (2021).
2. Pan, G. et al. Bright red YCl_3_-promoted CsPbI_3_ perovskite nanorods towards efficient light-emitting diode. *Nano Energy* **81**, 105615 (2021).
3. Meng, C. et al. Synthesis of single CsPbBr_3_@SiO_2_ core-shell particles via surface activation. *Journal of Materials Chemistry C* **8**, 17403-17409 (2020).
4. Zheng, Y. et al. Effect of polymethyl methacrylate on in situ patterning of perovskite quantum dots by inkjet printing. *Luminescence* **39**, e4691 (2024).

5. Yang, Z. et al. Single-nanowire spectrometers. *Science* **365**, 1017 (2019).
